# Spider venom-derived peptide JZTX-14 prevents migration and invasion of breast cancer cells *via* inhibition of sodium channels

**DOI:** 10.3389/fphar.2023.1067665

**Published:** 2023-03-23

**Authors:** Wenfang Wu, Yuan Yin, Peihao Feng, Gong Chen, Liangyu Pan, Panyang Gu, Siqin Zhou, Fulong Lin, Siyu Ji, Chunbing Zheng, Meichun Deng

**Affiliations:** ^1^ Department of Biochemistry and Molecular Biology and Hunan Province Key Laboratory of Basic and Applied Hematology, School of Life Sciences, Central South University, Changsha, Hunan, China; ^2^ Hunan Key Laboratory of Animal Models for Human Diseases, Hunan Key Laboratory of Medical Genetics, School of Life Sciences, Central South University, Changsha, Hunan, China; ^3^ Xiangya School of Medicine, Central South University, Changsha, Hunan, China; ^4^ Hunan Yuanpin Cell Technology Co. Ltd, Hunan, China

**Keywords:** JZTX-14, nav1.5, TNBC, migration, transcriptomics

## Abstract

Nav1.5 channel is crucial for the proliferation and migration of breast cancer cells. In this study, we investigated the anticancer effect of JZTX-14, a natural peptide considered an effective antagonist of Nav1.5. First, we successfully isolated and purified the 31 amino acid peptide JZTX-14 containing three pairs of disulfide bonds from spider venom and synthesised JZTX-14 by solid phase synthesis. We then predicted their physiochemical properties and structures in the peptide database. Further, we investigated the effects of natural and synthetic JZTX-14 on the proliferation and migration of MDA-MB-231 breast cancer cells *via* modulation of sodium current through the Nav1.5 channel. The results showed that both synthetic and natural JZTX-14 inhibited Nav1.5 currents, indicating the successful synthesis of JZTX-14. However, JZTX-14 did not affect MDA-MB-231 cell proliferation but inhibited its migration. Transcriptome analysis revealed that JZTX-14 downregulated S100A4 and FBXO2 and upregulated SERPINB2 in MDA-MB-231 cells. Western blot analysis demonstrated an increased level of the epithelial marker, E-cadherin, and decreased levels of the mesenchymal markers, N-cadherin and vimentin, and matrix metalloproteinase (MMP2), indicating the possible underlying mechanism of the inhibition of MDA-MB-231 cell migration by JZTX-14. This study provides a new target for inhibiting breast cancer metastasis and identifies a potent natural peptide for treating Triple-negative breast cancer.

## 1 Introduction

Cancer, one of the most life-threatening diseases, is a global public health problem, especially in China and other developed countries. (Bray et al., 2021). Statistics on cancer incidence in the United States show that in 2023, breast cancer will be the most common cancer among women, ranking second in female cancer-related mortality worldwide after lung cancer (Siegel et al., 2023). Clinically, based on the expression of molecular indicators, such as estrogen (ER), progesterone (PR), human epidermal growth factor receptor 2 (HER2), and Ki-67, breast cancer is divided into the following types: luminal A, luminal B (HER2-negative), luminal B (HER2-positive), and HER2-positive (Yeo and Guan, 2017). Triple-negative breast cancer (TNBC) is a subtype of breast cancer characterised by the negative expression of ER, PR, and HER2 (Harman et al., 2013). Notably, in clinical practice, TNBC usually shows a strongly aggressive nature and lacks therapeutic options (Nedeljković and Damjanović, 2019). Moreover, the high prevalence is accompanied by an extremely short survival time in patients with TNBC (Dent et al., 2007; Gobbini et al., 2018). Metastasis is the leading cause of death in triple-negative breast cancer and the most critical therapeutic target (Zhao et al., 2020). Therefore, there is an urgent need to develop novel therapeutic approaches and targeted drugs.

Voltage-gated sodium channels (Na_v_s; VGSCs) are membrane proteins with nine isoforms responsible for the rapid upstroke of action potentials in excitable cells (de Lera Ruiz and Kraus, 2015). VGSCs are present in excitable and non-excitable cells, including microglia, astrocytes, immune cells, fibroblasts, and cancer cells, and play various biological roles, such as tumour cell migration and immune cell phagocytosis (Black and Waxman, 2013; Roger et al., 2015). Nav1.5, encoded by SCN5A, is an essential receptor in breast cancer growth and migration (Rajaratinam et al., 2022). Notably, inhibition of Nav1.5 function or expression can inhibit breast cancer cell migration *in vivo* and *in vitro* (Brackenbury et al., 2007; Nelson et al., 2015). Downregulation of Nav1.5 expression in an orthotopic breast cancer model significantly reduced tumour growth, local invasion of surrounding tissues, and metastasis of the liver, lung, and spleen. Downregulation of Nav1.5 did not affect cell proliferation or angiogenesis in tumours but increased cell apoptosis. *In vitro*, downregulated Nav1.5 changed the cell morphology and reduced the expression of CD44 (Brackenbury et al., 2007; Nelson et al., 2015). DHA inhibits Nav1.5 current and NHE-1 activity in human breast cancer cells, reducing the invasiveness of Nav1.5-dependent cancer cells (Wannous et al., 2015). Phenytoin inhibited the transient and persistent Na^+^ current in strongly metastatic MDA-MB-231 cells, thus significantly inhibiting the migration and invasion of MDA-MB-231 cells (Yang et al., 2012). A recent report showed that the size and mass of breast tumour tissue were reduced after treatment with Nav1.5 antibody in triple-negative breast cancer cells MDA-MB-231 (Sharudin et al., 2022). Nav1.5 mRNA and protein are highly overexpressed in breast tumours compared with normal tissues and are associated with cancer recurrence, metastasis, and a low survival rate. In breast cancer cells, Na^+^ influx mediated by non-inactivated Nav1.5 channels allosterically increases the activity of the Na^+^-H^+^ exchanger NHE1, thereby promoting the efflux of H^+^ and further increasing Na^+^ into cancer cells, subsequently alkalizing intracellular pH and reducing extracellular pH (Brisson L, et al., 2011). Acidifying the microenvironment surrounding cells favours the activity of extracellular proteases that digest the extracellular matrix, such as acid cysteine cathepsin, thereby allowing cancer cells to invade the extracellular matrix. In addition, Nav1.5 activity maintains Src kinase activity, actin polymerisation, and the acquisition of a spindle-shaped elongated morphology of cancer cells (Brisson L et al., 2013). In conclusion, Nav1.5 plays a critical role in “mesenchymal invasion”. Nav1.5 maintains the expression of the transcription factor SNAI1 by EMT, obtains mesenchymal phenotypes, and enhances invasion (Gradek et al., 2019). Ranolazine inhibits Nav1.5-mediated breast cancer cell invasiveness and lung colonisation (Driffort et al., 2014). In summary, Nav1.5 is a potent therapeutic target for TNBC (Luo et al., 2020). However, the mechanism by which Nav1.5 affects breast cancer cell migration remains unclear.

JZTX-14, a venom from the spider *Chilobrachys jingzhao*, effectively inhibits the function of HEK293 cells heterologously expressing Nav1.5 (Zhang et al., 2018); however, it is unclear how it affects tumour cells, especially malignant TNBC cells. In our study, we found that both natural and synthetic JZTX-14 inhibited Nav1.5, indicating that successfully synthesised JZTX-14 could be used as an effective antagonist of the Nav1.5 channel. JZTX-14 significantly inhibited sodium currents in MDA-MB-231 cells. JZTX-14 did not affect MDA-MB-231 cell proliferation but significantly inhibited the migration of MDA-MB-231 cells.

## 2 Materials and methods

### 2.1 Peptide synthesis

The solid-phase chemical synthesis of peptides was carried out on a peptide synthesizer using a solid-phase peptide chemical synthesis method coupled with Fmoc-amino acids and HCTU. The crude polypeptide was separated and purified by reverse-phase HPLC (reverse-phase column: 4.6 mm × 250 mm Sinochrom ODS-BP five column; eluent A: acetonitrile containing 0.1% TFA; eluent B: aqueous solution containing 0.1% TFA, the concentration change of solution A within 25 min is 32%–57%; the flow rate is 1.0 ml/min). The mass-to-charge ratio of each peak was analyzed by MALDI-TOF/TOF mass spectrometry to determine the position of the target peak, and then collected target peak was freeze-dried under vacuum. The target peak used analytical reverse-phase HPLC for separation and purification. The above separation and purification ensure that the purity of the synthesized product reaches more than 95%, and then freeze-dried for use.

### 2.2 Peptide renaturation

Take 0.1 mg of purified synthetic crude peptides for oxidative renaturation in 1 ml of 0.1 mol/L Tris-HCl, a certain concentration of GSH/GSSG, and buffers with different pH values. For each renaturation condition, 100 μl of the reaction solution was taken out at different time intervals, and 10 μl of 50% TFA aqueous solution was added to terminate the reaction, and then the oxidative renaturation was monitored in real time by analytical reverse-phase HPLC, and the collected elution peaks were subjected to mass spectrometry. Analysis to determine the appropriate renaturation time and pH, and the optimal conditions for oxidative renaturation of the native toxin.

### 2.3 Physicochemical properties and structure prediction of JZTX-14

Bioinformatics analysis of JZTX-14 was performed using ProtParam software in ExPASy (https://web.expasy.org/), physicochemical parameters including: theoretical isoelectric point, amino acid and atomic composition analysis, half-life, GRAVY index (hydrophilic (grand average of sex), lipid solubility index, instability index, etc. The hydrophobicity of JZTX-14 was predicted using Protscale software. Potential splice sites for JZTX-14 by trypsin and other enzymes were predicted using PeptideCutter software. The online software I-TASSER (http://zhanglab.ccmb.med.umich.edu/I-TASSER/) was used structure prediction.

### 2.4 Whole-cell recording

Sodium currents in experimental cells were recorded at room temperature (22°C–25°C) using an EPC-10 patch-clamp amplifier (HEKA, Germany) in a whole-cell patch-clamp configuration. Recording pipettes were made from borosilicate glass capillaries (VWR, United States) using a PC-10 puller (Narishige, Japan). The inner solution (mM): KCl 130, NaCl 15, CaCl_2_ 0.37, MgCl_2_ 1 EGTA 1, HEPES 10, pH 7.2, resistance was measured as 1.8 MO. The outer solution was composed of (mM): NaCl 140, KCl 3, MgCl_2_ 1, CaCl_2_ 1, HEPES 10, pH 7.2. To minimize voltage errors, the series resistance compensation was set to 80%. The P/4 protocol was used to subtract the linear capacitance and leakage current. Experimental data were acquired and analyzed using the pulse fit 8.0 program (HEKA, Germany). The needed concentrations of toxin dissolved in external solution were applied to the cell surface by microinjector.

### 2.5 Cell culture

Breast cancer cells MDA-MB-231 were purchased from Procell Life Science and Technology (Wuhan, China) and used between the 10th passage and 30th passage. MDA-MB-231 cells were maintained in Dulbecco’s modified Eagle’s medium F12 (DMEM/F12) supplemented with 10% fetal bovine serum (PAN, Australian origin) and 1% Penicillin/Streptomycin (Sangon Biotech, Shanghai, China). All cells were incubated at 37°C under 5% CO2 in incubator.

### 2.6 Cell counting Kit-8 (CCK-8) assay

Cells were seeded in 96-well plates, incubated for 12 h and then replaced with complete DMEM/F12 medium containing different concentrations of inhibitors for 24 h. Cell proliferation was evaluated by CCK-8 (TargetMol, Shanghai, China) analysis according to the manufacturer’s instructions. The optical density (OD) was recorded at 450 nm.

### 2.7 Scratch wound assay

The MDA-MB-231 cells were seeded into a six-well plate at an appropriate density to form a single layer of cells. After 12 h, a sterile 200 μl pipette tip was used to produce a linear scraping wound, which was cultured with DMEM/F12 containing 0.2% BSA and different concentrations of inhibitors. The cells were imaged at 0 h, 24 h and 48 h, after scratch induction. ImageJ software was used to analyze the images and quantify cell migration.

### 2.8 Transwell for cell migration assay

Cell migration assay was performed using 24-well transwell plate (Corning, United States). 5×10^4^ cells/well were seeded in the upper chamber in DMEM/F12 medium containing 0.1% BSA, the lower chamber contained DMEM/F12% and 10% BSA. After 24 h incubation, the cells on the filter membrane were washed three times with PBS, then fixed with 4% paraformaldehyde for 30 min and stained with 0.1% crystal violet for 20 min. The cells on the filter were observed by microscopy and the number of cells was determined using the quantification software ImageJ.

### 2.9 Hoechst 33342/PI double staining assay

The cells were seeded in a 6-well plate, and after their adherence to the plate, the medium was changed with a complete medium containing different concentrations of inhibitors. After 48 h of treatment, the cells were washed three times with 4°C cold PBS, and incubated with 5 μl/ml Hoechst 33,342 for 15 min. The cells were washed again three times using PBS and incubated with 5 μl/ml PI for 15 min. Finally, the stained cells were observed with a fluorescence microscope (Zeiss, Germany).

### 2.10 General transcriptome library construction and sequencing

Total RNA was extracted from untreated (C) MDA-MB-231 cells and MDA-MB-231 cells treated with a concentration of 12.5 μM JZTX-14 (GL) for 48 h using TRIzol reagent. RNA concentration was first quantified using a NanoDrop 2000 spectrophotometer and further accurately quantified using an Agilent 2100/4200 for RNA sample mass and concentration. The extracted total RNA was stored at −70°C. The sample mRNA was enriched with magnetic beads with Oligo (dT), and then the mRNA was broken into short fragments which subsequently used as a template to synthesize the first strand of cDNA with six-base random primers. The second cDNA strand was synthesized by adding buffer, dNTPs, and enzymes, and the acquired double-stranded cDNA was purified, end-repaired, A-tailed, and connected to sequencing junctions. Next, the fragment size was selected and the cDNA library was enriched by PCR amplification, followed by accurate qualification of the effective concentration of the library using qPCR. After library quality control, PE150 sequencing was performed using Illumina Novaseq6000 platform (BerryGenomics, Beijing, China).

### 2.11 Mapped reads statistics and comparison with reference genome

The reads after rRNA removal were compared with the reference genome using Hisat2 software. Gene level quantification was performed separately for each sample using FeatureCount and combined to obtain the gene expression matrix of all samples. Correlation coefficients between samples were calculated based on the expression values (FPKM) of genes in each sample. Differential expression significance analysis was performed using edgeR with |log2(Fold Change)|> 0.585 and *p*-value < 0.05 as parameters to obtain the list of different genes for different comparative combinations.

### 2.12 Functional annotation of DEGs and enrichment analysis

Gene Ontology (GO) is an international standardized classification system for gene functions, which is divided into three major categories: Biological Process, Molecular Function and Cellular Component, which are used to describe the biological processes, molecular functions and cellular environments in which the products encoded by genes are involved respectively. They are used to describe the biological process, the molecular function and the cellular environment in which the gene encoded product is located. The GO enrichment analysis of differentially expressed genes was performed using topGO software, and the number of genes in each GO term that was significantly enriched was counted and classified into secondary categories. The number of genes in each GO term that was significantly enriched was counted, and the secondary classification statistics were performed. KEGG (Kyoto Encyclopedia of Genes and Genomes) is a systematic analysis of gene functions, genomic information database, and study of gene and expression information as a whole network. KEGG analysis aims to find metabolic pathways associated with target genes in terms of biochemical metabolic pathways, etc. KEGG enrichment analysis was performed using KOBAS (v3.0) software.

### 2.13 Quantitative real-time PCR

Total RNA was extracted with TRIZOL (Vazyme; Nanjing, China), and 2 ug of total RNA was reverse transcribed to synthesize cDNA under the following conditions 25 °C for 5 min; 37°C for 45 min; 85°C for 5 s. The target genes were quantified using ChamQ Universal SYBR qPCR Master Mix (Vazyme; Nanjing, China) in Bio-Rad Real-Time Quantitation System (United States). The primer sequences were synthesized by Sangon Biotech with the following sequences: ANGPTL4 Forward 5′- CTC​TGG​AGG​CTG​GTG​GTT​TG-3′ Reverse5′- AGA​GTC​ACC​GTC​TTT​CGT​GG-3’; S100A4 Forward 5′-TCT​CTC​CTC​AGC​GCT​TCT​TC-3′ Reverse5′- GCT​GTC​CAA​GTT​GCT​CAT​CA-3’; GJB3 Forward 5′-CCC​AAC​ATC​GTG​GAC​TGC​TA-3′ Reverse5′- GGC​GCC​CAC​CAT​GAA​GT-3’; FBXO2 Forward 5′- GTG​TCG​CAA​AGC​ACA​GGT​C-3′ Reverse5′- CGG​ACA​GTA​GCT​TAA​CGG​TGA​G-3’; PLAC1 Forward 5′-AAA​TTT​GGC​AGC​TGC​CTT​CAC-3′ Reverse5′- TGA​TGC​CAC​ATT​CAG​TAA​CAC-3′ HMGN5 Forward 5′-TCG​GCT​TTT​TTT​CTG​CTG​ACT​AA-3′ Reverse5′- CTC​TTT​GGC​TCC​TGC​CTC​AT-3’; IL-24-F Forward 5′-TTG​CCT​GGG​TTT​TAC​CCT​GC-3′ Reverse5′- AAG​GCT​TCC​CAC​AGT​TTC​TGG-3’; SERPINB2 Forward 5′-GCA​TGT​TCT​TGT​TGC​TTC​CA-3′ Reverse5′- TTC​AGC​CAT​TTT​GTC​TTT​GC-3’; GAPDH Forward 5′-GGA​GCG​AGA​TCC​CTC​CAA​AAT-3’; Reverse5′-GGCTGTTGTCATACTTCTCATGG-3’.

### 2.14 Western blot analysis

Cells treated with different concentrations of JZTX-14 were lysed with RIPA lysis solution and samples were collected. Protein concentrations were determined by BCA assay kit (Biosharp, China) and denatured by adding SDS loading buffer (Bio-Bad, United States) followed by boiling. Equal amounts of total protein lysates were subjected to SDS-PAGE and transferred to PVDF membrane (Merck Millipore, United States), 5% BSA was used for blocking (1 h). Primary antibody (Nav1.5, E-cadherin, GAPDH and Vimentin antibody for Proteintech; N-cadherin for Immunoway) was incubated overnight at 4°C, secondary antibody (Abbkine, China) was incubated for 1 h at room temperature and the membrane was visualized by chemiluminescence.

### 2.14 Statistical analysis

Statistical comparisons were performed using one-way analysis of variance (ANOVA) and values of *p* < 0.05 were considered significant. For multiple comparison between groups, Dunnet *t*-test was performed after one-way ANOVA in all pertinent experiments. Data are expressed as mean ± SEM and representative of at least three independent experiments.

## 3 Results

### 3.1 Purification and characterisation of JZTX-14

#### 3.1.1 Solid phase chemical synthesis of JZTX-14

**FIGURE 1:**
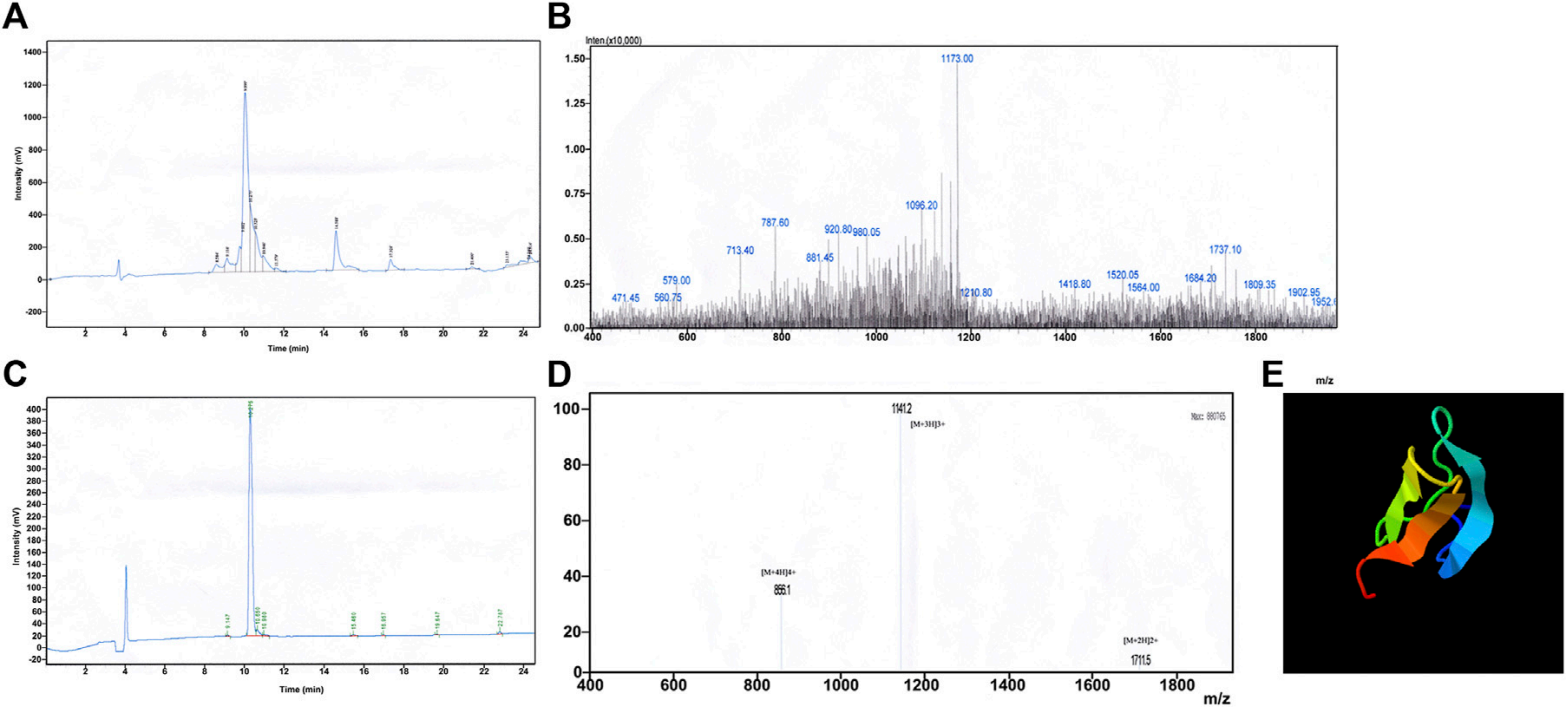
Synthesis and redox refolding of peptides. **(A)** Reversed-phase HPLC profile after peptide synthesis; **(B)** Identification of synthetic peptides by MALDI-TOF/TOF mass spectrometry; **(C)** Reversed-phase HPLC profile of the peptide after renaturation; **(D)** Identification of peptides after refolding by MALDI-TOF/TOF mass spectrometry; **(E)** 3D structure prediction by I-TASSER.

The synthesised crude JZTX-14, obtained as a white powder after vacuum freeze-drying, was separated and purified by RP-HPLC (Figure 1A). The obtained elution peaks were identified by MALDI-TOF/TOF mass spectrometry, where the relative molecular weight (MH^+^) was 3427.09 (Figure 1B), consistent with the theoretical molecular weight of 3422.17 of JZTX-14 (linear), suggesting that the solid-phase peptide synthesis was successful.

#### 3.1.2 Redox renaturation of peptides

Chemically synthesised JZTX-14 is a linear molecule without biological activity; therefore, oxidative renaturation must be carried out before detecting the activity to form the correct spatial structure and disulfide bond pairing. According to the results of previous renaturation studies, the optimal oxidative renaturation conditions are as follows: the mass concentration of the sample is 0.05 g/L, containing 1.0 mM/L GSH, 0.1 mM/LGSSG, 0.1 M/L Tris-HCl buffer, pH 8.0, and renaturation temperature 4°C. Figure 1C shows oxidative renaturation of linear JZTX-14 (Figure 1C). The product was identified by MALDI-TOF/TOF mass spectrometry with a relative molecular mass of 3421.09 (Figure 1D), lower than that of linear JZTX-14 by 6, suggesting that the renatured JZTX-14 formed three pairs of disulfide bonds.

#### 3.1.3 Physicochemical properties and structure prediction of JZTX-14

ProtParam analysis showed that JZTX-14 contains 31 amino acid residues, with a molecular formula of C_153_H_224_N_38_O_40_S_6_ and a total of 461 atoms. Parameters such as molecular weight, isoelectric point, total hydrophobic ratio, total net charge, Wimley–White whole-residue hydrophobicity (the sum of whole-residue free energy of transfer of the peptide from water to the POPC interface), number of charged amino acids, half-life, stability, hydrophobicity, and hydrophilicity are listed in Table 1. The protein was considered unstable when the instability index was greater than 40. The contents of aliphatic amino acids alanine, valine, leucine, and isoleucine in a polypeptide can determine its lipid solubility index. The fat index reflects the thermal stability of proteins and peptides and can determine the depth of the hydrophobic surface, thereby affecting the hydrophobicity of the peptides. The test results also demonstrated the general thermal stability and long half-life of JZTX-14. Generally, the grand average of hydropathicity (GRAVY) value ranges from −2 to +2, with positive values indicating hydrophobic proteins. GRAVY of the JZTX-14 polypeptide was 0.294, indicating that the protein was hydrophobic.

**TABLE 1:** Physicochemical properties of JZTX-14. Predicted by ProtParam.

Physical and chemical properties	Parameter
Number of amino acids	31
Molecular weight/Da	3428.05
pI	6.7
Total hydrophobic ratio	48%
The total net charge	+0.25
The Wimley-White whole-residue hydrophobicity	−2.25
Total number of negatively charged residues	1
Total number of positively charged residues	1
Estimated half-life in mammalian reticulocytes, vitro/h	30 h
Estimated half-life in yeast, vivo/h	>20 h
Estimated half-life in Escherichia coli, vivo/h	>10 h
Instability index	60.46
Aliphatic index	53.55
GRAVY	0.294

The online analysis using the ProtScale tool found (calculated by Kyte and Doolittle) that the maximum hydrophobic index of JZTX-14 was 1.144, located at the 20th amino acid, and the minimum hydrophobic index was −1.222, located at the 11th amino acid. This result suggests that JZTX-14 is a hydrophobic protein, consistent with its physicochemical properties.

PeptideCutter software analysis showed that most of the amino acid sites in JZTX-14 could be cleaved by 16 active substances (Table 2). Enzymes with more cleavage sites are Proteinase K, Pepsin (pH > 2), pepsin (pH 1.3), and thermolysin. The 3D structure prediction results obtained using the online prediction software, I-TASSER, are shown in Figure 1E.

**TABLE 2:** The predicted shear sites of JZTX-14.

Name of enzyme	No. of cleavages	Positions of cleavage sites
Asp-N endopeptidase + N-terminal Glu	1	26
BNPS-Skatole	2	7 24
Chymotrypsin-high specificity (C-term to [FYW], not before P)	3	5 6 7
Chymotrypsin-low specificity (C-term to [FYWML], not before P)	5	5 6 7 20 31
Glutamyl endopeptidase	1	27
Iodosobenzoic acid	2	7 24
LysC	1	4
LysN	1	3
NTCB (2-nitro-5-thiocyanobenzoic acid)	6	1 8 15 16 21 28
Pepsin (pH1.3)	6	4 5 19 20 30 31
Pepsin (pH > 2)	8	4 5 7 19 20 24 30 31
Proline-endopeptidase [*]	1	11
Proteinase K	13	5 6 7 8 20 21 23 24 26 27 28 30 31
Staphylococcal peptidase I	1	27
Thermolysin	6	4 5 19 20 29 30
Trypsin	1	4

### 3.2 Comparison of the effects of natural (nJZTX-14) and synthetic JZTX-14 (sJZTX-14) on Nav1.5 channels heterologously expressed in HEK293T cells

**FIGURE 2:**
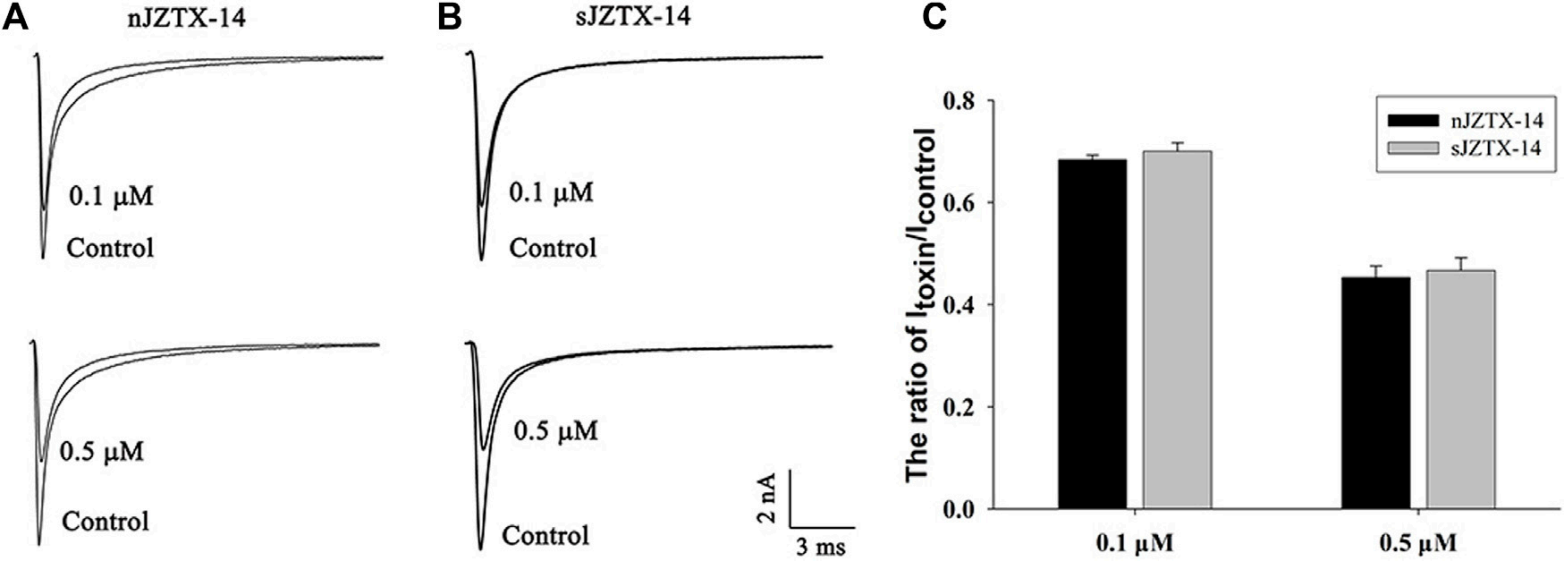
Comparison of sodium currents inhibited by natural and synthetic JZTX-14 in HEK293T cells heterologously expressing Nav1.5 **(A)** Currents inhibited by natural JZTX-14; **(B)** Currents inhibited by synthetic JZTX-14; **(C)** Ratio of the remaining sodium currents after toxin treatment

To determine the biological activity of synthetic and natural JZTX-14 on Nav1.5, we observed their effects on HEK293T cells heterologously expressing Nav1.5 using a whole-cell voltage-clamp configuration. The current traces were induced by a 50 ms depolarisation potential of −30 mV at a holding potential of −100 mV. Currents were recorded by adding 0.1 μM and 0.5 μM of JZTX-14 to the solution. Natural JZTX-14 inhibited 31.7% ± 1.3% and 55.1% ± 2% currents at 0.1 μM and 0.5 μM, respectively (Figures 2A, C). Synthetic JZTX-14 inhibited 30.3% ± 2.5% and 53.4% ± 2.5% currents at 0.1 μM and 0.5 μM, respectively (Figures 2B, C). Our data demonstrated that natural and synthetic JZTX-14 might have the same biological activities on Nav1.5 channels.

### 3.3 Effects of JZTX-14 on MDA-MB-231 sodium channels

**FIGURE 3:**
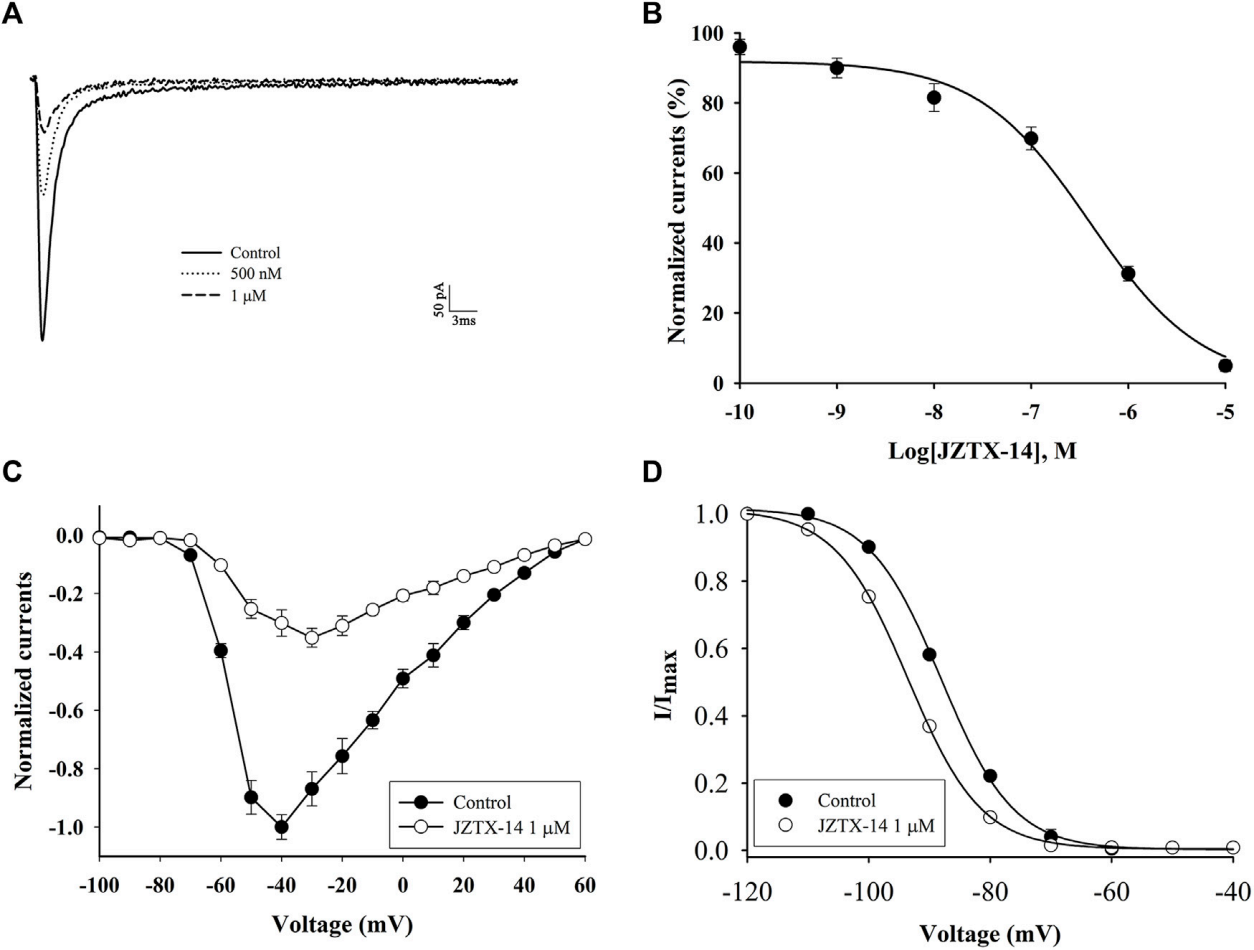
JZTX-14 inhibited the sodium current in MDA-MB-231 cells. **(A)** Inhibition of sodium current by 500 nM and 1 μM JZTX-14; **(B)** Dose-dependent inhibition of sodium current by 1 μM JZTX-14; **(C)** The current-voltage (I–V) curves of sodium currents show the average relationship of current trajectories before and after toxin treatment. The series of current trajectories before and after adding 1 μM JZTX-14 at 50 ms depolarisation at a holding potential of −100 mV to various potentials. **(D)** Inactivation kinetics inhibition of sodium currents by 1 μM JZTX-14. Data points (6 cells per point) show currents relative to control current amplitudes.

The biological activity of synthetic JZTX-14 in MDA-MB-231 cells was recorded using the whole-cell patch-clamp method. MDA-MB-231 sodium channels are sensitive to JZTX-14. Both 500 nM and 1 μM JZTX-14 inhibited the inward sodium currents. The inward sodium current was induced by a 50 ms depolarisation potential of −30 mV at a holding potential of −100 mV (Figure 3A). The inhibition by JZTX-14 was concentration-dependent (Figure 3B). Figure 2B shows the fitting of dose-response curves with the Hill equation, which yielded IC_50_ values of 401 nM for the MDA-MB-231 sodium channels. Next, we determined the effects of JZTX-14 on the current-voltage (I–V) curves of sodium channels resulting from a series of currents induced by a set of depolarising potentials ranging from −100 to +60 mV when cells were held at −100 mV (Figure 3C). As shown in Figure 3C, the activation thresholds shifted by approximately 10 mV in the depolarising direction. We also investigated the effect of JZTX-14 on the steady-state inactivation of sodium channels in MDA-MB-231 cells. JZTX-IX shifted the inactivation curves for sodium channels by approximately 10 mV (Figure 3D).

### 3.4 Effect of JZTX-14 on the proliferation and migration of MDA-MB-231 cells

**FIGURE 4:**
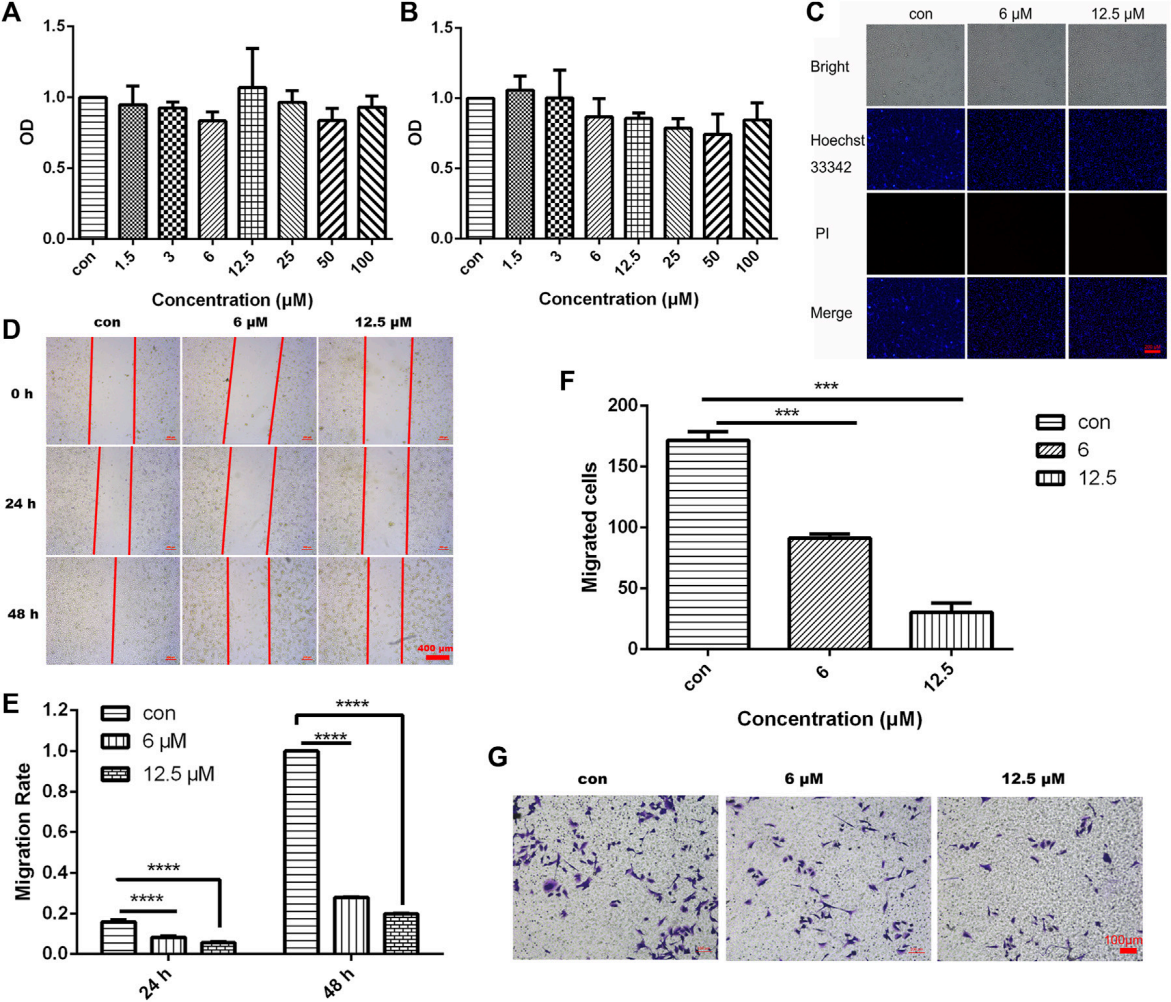
JZTX-14 had no effect on the proliferation of MDA-MB-231 cells but inhibited cell migration and invasion. **(A,B)** MDA-MB-231 cells were treated with different concentration gradients of JZTX-14 for 24 and 48 h, and cell viability was assessed using CCK-8 assay. **(C)** JZTX-14 (0, 6, and 12.5 μM) had no significant effect on the necrosis of MDA-MB-231 cell lines as analyzed using Hoechst 33,342 and PI staining; PI (red) for necrotic cell nuclear staining and Hoechst 33,342 (blue) for nuclear staining. Barcodes represent 200 μm; **(D,E)** Representative images and statistical analysis of MDA-MB-231 cells in the wound healing assay after treatment with JZTX-14 (0, 6, and 12.5 μM). **(F,G)** Representative images and statistical analysis of MDA-MB-231 cells using transwell assay after treatment with JZTX-14 (0, 6, and 12.5 μM).

To investigate the effect of different concentrations of JZTX-14 on the proliferation of MDA-MB-231 cells, we treated MDA-MB-231 cells with 1.5, 3, 6, 12.5, 25, 50, and 100 μM JZTX-14 for 24 and 48 h and then assayed the cellular activity of each group using the CCK-8 assay (Figures 4A, B). The results showed that different concentrations of JZTX-14 did not affect the survival rate of MDA-MB-231 cells after 24 h of treatment, and JZTX-14 at 25, 50, and 100 μM concentrations slightly reduced the survival rate of cells after 48 h of treatment. However, the difference was not statistically different compared to the control group, indicating that JZTX-14 had little effect on the survival rate of MDA-MB-231 cells. Hoechst 33,342 and PI dye staining results indicated no apoptosis and necrosis of MDA-MB-231 cells after treatment with JZTX-14 (0, 6, 12.5 μM) (Figure 4C). The scratch assay results showed that 12.5 μM JZTX-14 inhibited the migration of MDA-MB-231 cells by 72% after 24 h and 80% after 48 h (Figures 4D, E). Transwell assay indicated that JZTX-14 inhibited cell invasion in a dose-dependent manner, with 50% and 75% inhibition at 6 and 12.5 μM, respectively (Figures 4F, G).

### 3.5 Data quality for RNA sequencing and differentially expressed gene analysis

**FIGURE 5:**
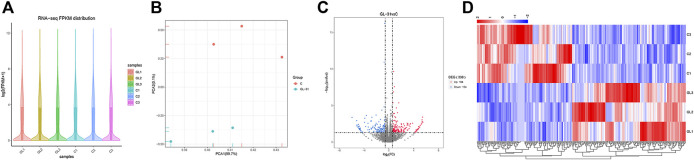
RNA-seq data analysis. **(A)** Distribution of gene expression levels of each sample (GL1, GL2, GL3 and C1, C2, C3); **(B)** PCA was performed on samples; **(C)** The volcano map mapped 338 differential genes, including 154 downregulated and 184 upregulated genes; **(D)** Heat map of differentially expressed gene clustering. Heat map colour represents the level of gene expression; red indicates high expression, and blue indicates low expression.

Six samples were sequenced to obtain raw data and assess data quality. The results showed that all six sequenced samples met the requirements for subsequent data analysis, based on the violin plot of FPKM values of all genes in each sample showing the distribution of gene expression abundance in each sample (Figure 5A). The results of principal component analysis (PCA) showed that the transcriptome data could distinguish the control group (C1, C2, and C3) from the JZTX-14 group (GL1, GL2, and GL3) (Figure 5B). By comparing the gene expression profile data of groups C and GL (C/GL), 338 differentially expressed genes (DEGs), including 184 upregulated genes and 154 downregulated genes, were screened according to the threshold criterion of |log2 fold change| > 0.585 and *p* < 0.05. All DEGs are displayed in a volcano plot (Figure 5C). Bivariate hierarchical clustering analysis was performed on the selected differentially expressed genes and each group of samples, and the clustering results are shown in the heat map (Figure 5D).

### 3.6 Functional enrichment analysis

**FIGURE 6:**
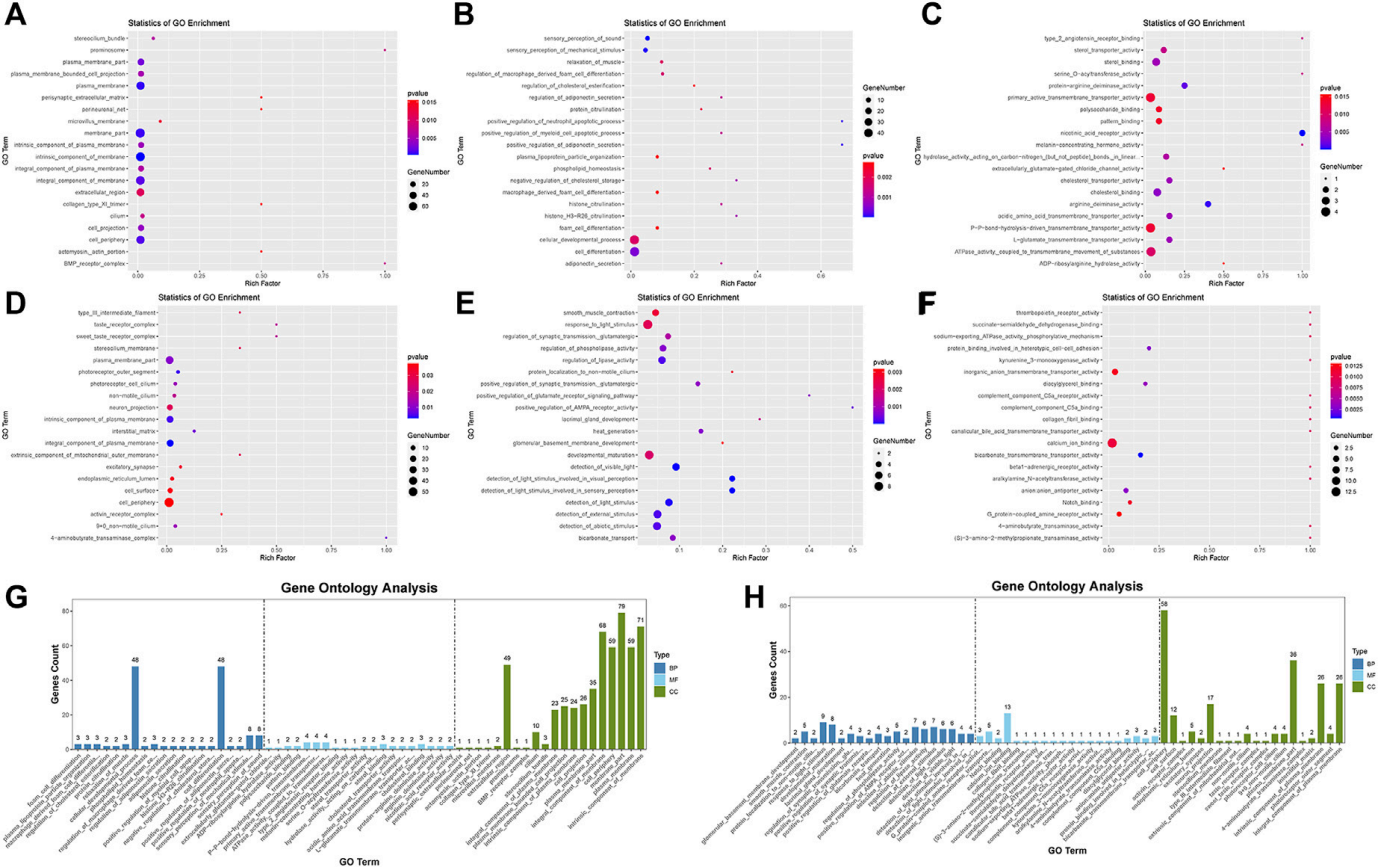
Results of gene ontology enrichment analysis. **(A,B,C)** Scatter plots show the top 20 significantly enriched gene ontology (GO) terms for the top 20 downregulated genes in three different functional groups of cellular components (CC), biological processes (BP), and molecular functions (MF) after JZTX-14 treatment of MDA-MB-231 cells. **(D,E,F)** Scatter plots show the top 20 significantly enriched gene ontology (GO) terms for the top 20 upregulated genes in CC, BP, and MF after JZTX-14 treatment of MDA-MB-231 cells. **(G)** GO terms for the top 20 significantly enriched downregulated genes in three different functional groups. **(H)** GO terms for the top 20 significantly enriched upregulated genes in three different functional groups. Dark blue indicates BP, light blue indicates MF, and green indicates CC.

**FIGURE 7:**
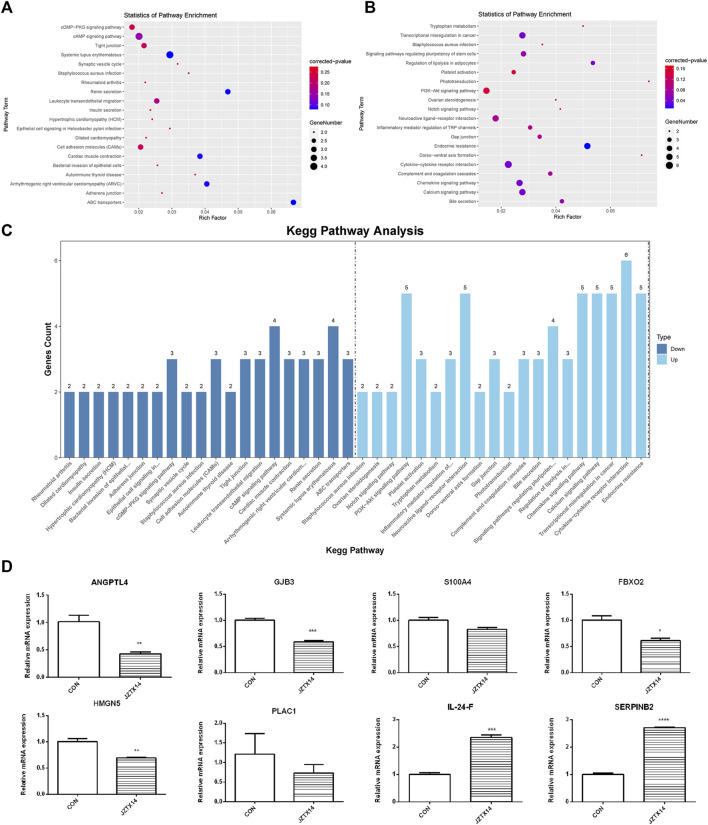
Results of KEGG pathway enrichment analysis and qPCR validation of migration-associated differential genes. **(A)** The top 20 significantly downregulated KEGG pathways in JZTX-14 -treated MDA-MB-231 cells. **(B)** The top 20 significantly upregulated KEGG pathways in JZTX-14 -treated MDA-MB-231 cells. **(C)** Number of genes in the top 20 pathways significantly enriched in downregulated and upregulated genes. **(D)** Significantly different migration and invasion-related genes in RNA sequencing results of JZTX-14-treated MDA-MB-231 cells were verified by qPCR. *ANGPTL4, GJB3, S100A4, FBXO2, HMGN5,* and *PLAC1* were downregulated; *IL-24-F* and *SERPINB2* were upregulated.

We used topGO software for GO enrichment analysis of differentially expressed genes and counted the genes in each GO term significantly enriched. A total of 3125 GO terms for biological processes (BP), 396 GO terms for cellular components (CC), and 521 GO terms for molecular functions (MF) were obtained for downregulated genes; 3198 GO terms for BP, 333 GO terms for CC, and 502 GO terms for MF were obtained for upregulated genes. The bubble plots of the top 20 GO terms (Figures 6A–F) are shown for each downregulated and upregulated gene classification system. The number of genes in each GO term that were significantly enriched in downregulated and upregulated genes are shown in Figures 6G, H, respectively. Compared to the control, MDA-MB-231 cells treated with JZTX-14 were enriched in GO analysis of extracellular space, intracellular signal transduction, integral component of the plasma membrane, receptor binding, cell adhesion, G-protein coupled receptor signalling pathway, G-protein coupled receptor signalling pathway coupled to cyclic nucleotide second messenger, and an extrinsic component of cytoplasmic side of the plasma membrane. In addition, we found the following pathways using KEGG pathway analysis: ABC transporters, cell adhesion molecules (CAMs), cAMP signalling pathway, cytokine-cytokine receptor interaction, calcium signalling pathway, and transcriptional misregulation in cancer (Figures 7A–C). Six downregulated genes, *ANGPTL4*, *S100A4*, *GJB3*, *FBXO2*, *PLAC1*, and *HMGN5*, and two upregulated genes, *IL-24-F* and *SERPINB2*, associated with migration and invasion, were screened for qPCR validation (Figure 7D).

### 3.7 JZTX-14 inhibits epithelial-mesenchymal transition in MDA-MB-231 cells

**FIGURE 8:**
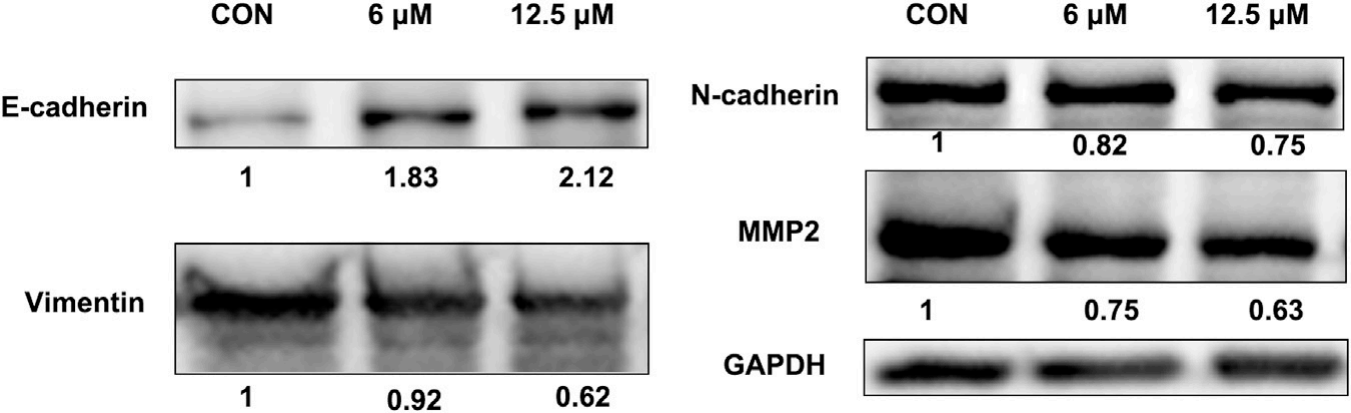
Western blot analysis to quantify expression levels of E-cadherin, N-cadherin, and vimentin in MDA-MB231 cells treated with different concentrations of JZTX-14.

Western blotting results showed that JZTX-14 significantly increased the expression levels of the epithelial marker E-cadherin and decreased those of the mesenchymal markers, N-cadherin and vimentin, and matrix metalloproteinase (MMP2) in MDA-MB231 cells compared to controls (Figure 8). Therefore, JZTX-14 may inhibit migration by regulating EMT.

## 4 Discussion

Spider venom is a rich natural product with significant antitumour effects in human lung adenocarcinoma, leukaemia, liver cancer, glioblastoma, and breast cancer (Gao et al., 2007; Liu et al., 2012; Lian et al., 2018; Barreto Dos Santos et al., 2019; Barreto et al., 2020; Lopez et al., 2020; de Avelar Júnior et al., 2022). We isolated and purified natural JZTX-14 from spider venom, which consists of 31 amino acids with three pairs of disulfide bonds. We also synthesised JZTX-14 by chemical synthesis and predicted its physicochemical properties and 3D structure using a peptide database. The effects of natural and synthetic JZTX-14 on sodium currents were examined in HEK293 cells heterologously expressing Nav1.5 at 0.1 and 1 μM, respectively. Both natural and synthetic JZTX-14 significantly inhibited Nav1.5 channels in a concentration-dependent manner, and thesimilar inhibition rate of natural and synthetic JZTX-14 on Nav1.5 at the same concentration indicates that the synthesised JZTX-14 was functionally identical to natural JZTX-14.

Spider venom and purified peptides affect cancer cells through five possible mechanisms: induction of cell cycle arrest, growth inhibition and apoptosis, inhibition of angiogenesis, inhibition of invasion and metastasis, and blockage of specific transmembrane channels (Rapôso, 2017). Nav1.5, a pore-forming alpha subunit of VGSC encoded by SCN5A, is not expressed in normal breast tissue or cells but is highly expressed in the breast cancer cell line MDA-MB-231 (Dutta et al., 2018). Elevated Nav1.5 expression promotes cell invasion and breakdown of extracellular matrix proteins, maintains actin polymerisation to produce F-actin stress fibres, and allows cancer cells to acquire a fibroblast morphology, suggesting a role for Nav1.5 in “mesenchymal invasion”. In orthotopic breast cancer, downregulation of Nav1.5 significantly inhibited tumour growth, local invasion of surrounding tissues, and metastasis to the liver, lung, and spleen but had no significant effect on tumour cell proliferation and angiogenesis. *In vitro* experiments, Nav1.5 downregulation changed the cell morphology and decreased the expression of CD44, indicating that VGSC activity may regulate cell invasion through the CD44-src-corticosteroid signal axis (Nelson M et al., 2015). Besides chemotherapy, there is no better treatment for TNBC. Therefore, there is an urgent need to develop novel drugs to reduce the recurrence and metastasis of cancer. Although CCK-8 and PI staining assays showed that JZTX-14 did not affect the proliferation and necrosis of MDA-MB-231 cells, scratching and transwell assays showed that JZTX-14 significantly inhibited the migration and invasion of MDA-MB-231 cells. BmKM9, derived from scorpion toxin peptide, is a nav1.5 inhibitor that significantly reduces the invasion and migration ability of MDA-MB231 cells but does not affect cell proliferation, cell cycle, and cell apoptosis. Similarly, the peptide FS50, derived from the flea *Xenopsylla cheopis*, is also a nav1.5 channel inhibitor that had no effect on TNBC proliferation but inhibits cell migration. TTX, a broad sodium channel inhibitor, inhibited cell migration and invasion but did not affect cell proliferation. This is consistent with a previous suggestion that VGSCS participate in cell motor activity without affecting proliferation (Roger et al., 2003; Gillet et al., 2009; Zhang et al., 2018). We hypothesised that inhibition of cell migration after JZTX-14 treatment might depend on the blockage of the Nav1.5 channel in MDA-MB-231 cells. Transcriptome analysis was performed to compare the differential genes in MDA-MB-231 cells after treatment with 0 and 12.5 μM JZTX-14. The 338 differential genes screened included 184 upregulated genes and 154 downregulated genes. GO enrichment analysis indicated the following enriched pathways: extracellular space, intracellular signalling, overall components of the plasma membrane, receptor binding, cell adhesion, G protein-coupled receptor signalling pathway, G protein-coupled receptor signalling pathway, coupling to cyclic nucleotide second messengers, and extrinsic components of the cytoplasmic side of the plasma membrane. KEGG enrichment showed differences between the control and JZTX-14 treatment groups in ABC transporters, cell adhesion molecules (CAMs), cAMP signalling pathway, cytokine-cytokine receptor interactions, and calcium signalling pathway. The results showed that JZTX-14 binding to Nav1.5 on MDA-MB-231 cells caused receptor changes that affected cell adhesion molecules and extracellular matrix, thereby inhibiting MDA-MB-231 migration. qPCR validated that the differentially significant genes *ANGPTL4, S100A4, GJB3, FBXO2, PLAC1, HMGN5, IL-24-F,* and *SERPINB2* were consistent with transcriptome trends. *S100A4, FBXO2,* and *SERPINB2* are associated with tumour EMT. EMT is associated with tumour progression and metastasis. In this process, epithelial cells transform into a migratory and invasive phenotype, with downregulated expression of epithelial markers, especially E-cadherin, and induced expression of mesenchymal markers (Kidd et al., 2014; Lamouille et al., 2014; Zheng and Kang, 2014). S100A4 is involved in the EMT process and considered a key molecular marker of EMT (Hua et al., 2016). S100A4 is a protein expressed in stromal cells. S100A4 promotes metastasis by inducing EMT. Knockdown of S100A4 expression in the breast cancer cell line MDA-MB-231 inhibited cell migration and invasion and significantly inhibited EMT. In addition, S100A4 knockdown decreased the expression of MMP2, a promoter and mediator of EMT in cancer. Most importantly, restoring the expression of MMP2 in MDA-MB-231 cells rescued the invasion ability of S100A4 knockdown inhibition and reversed the EMT of S100A4 knockdown inhibition. Previous studies have shown that the inhibition of S100A4 could inhibit the invasion ability of breast cancer cells through EMT and regulate MMP2 in breast cancer to participate in EMT (Xu et al., 2016). S100A4 is an upstream regulator of E-cadherin and vimentin expression. S100A4 knockdown increased E-cadherin expression and decreased vimentin expression. FBXO2 belongs to the F-box protein family and is a cytoplasmic protein and ubiquitin ligase F-box protein specific to hypermannose-glycoproteins. FBXO2 promoted the proliferation and migration of cancer cells, and FBXO2 knockdown inhibited the proliferation and migration of gastric cancer cells. Downregulation of FBXO2 decreased EMT in gastric cancer cells, increased E-cadherin expression, and decreased N-cadherin and vimentin expression (Sun et al., 2018). SERPINB2 is directly associated with developing multiple cancers and a poor prognosis. Downregulation of SERPINB2 is considered a part of EMT (Longhin et al., 2018). SERPINB2 o verexpression inhibits the aggressiveness and metastasis of liver and pancreatic cancers (Croucher et al., 2008). TXL promoted the increased expression of SERPINB2 in MDA-MB-231 cell and inhibited MDA-MB-231 cell migration (Nelczyk et al., 2022).

**FIGURE 9:**
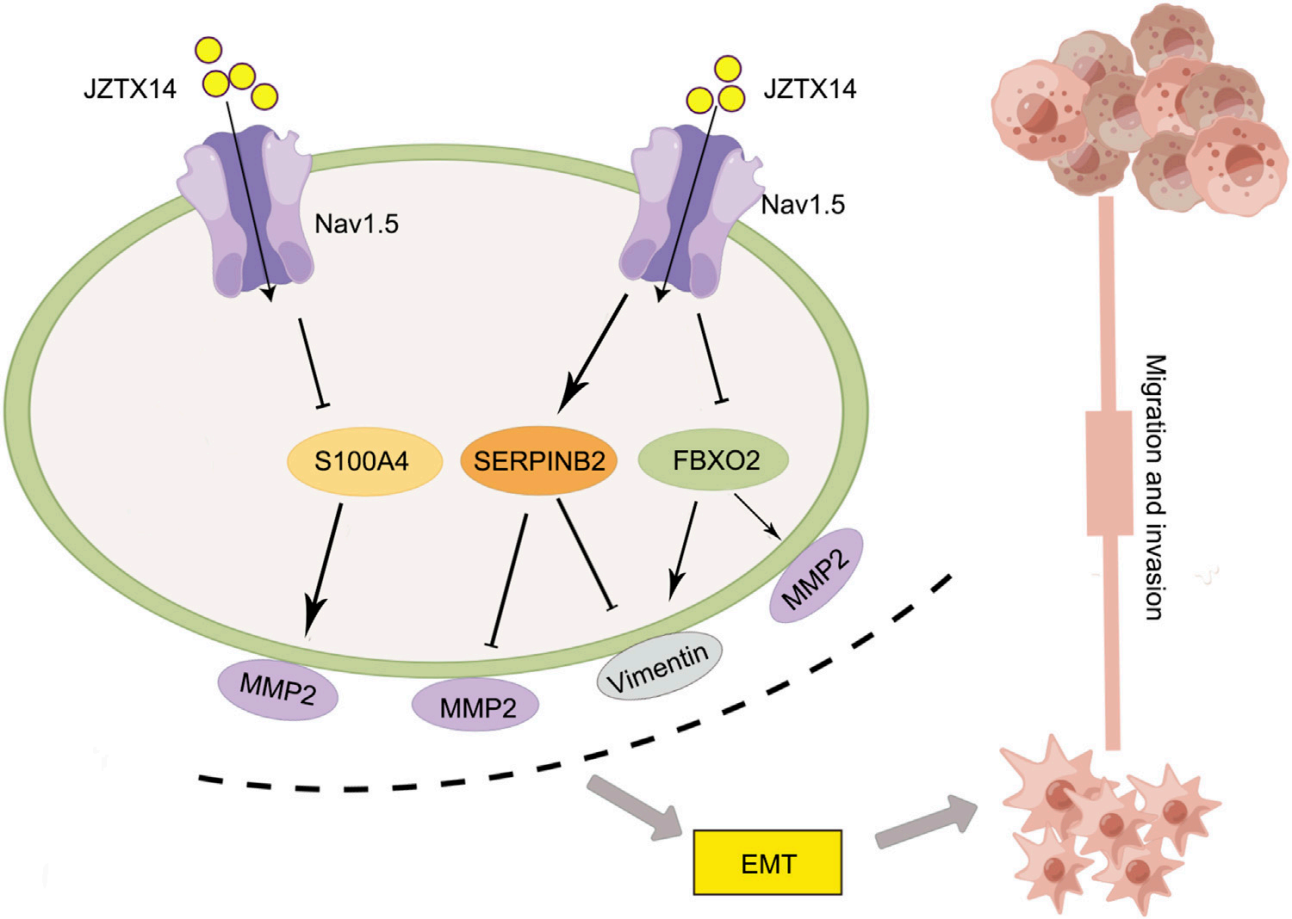
Schematic diagram of the molecular mechanism by which JZTX-14 inhibits the migration and invasion of MDA-MB-231 cells. The figure is drawn with the help of Figdraw.

Western blotting results showed that JZTX-14 significantly increased the expression levels of the epithelial marker E-cadherin and decreased those of the mesenchymal markers, N-cadherin and vimentin, and matrix metalloproteinase (MMP2) in MDA-MB231 cells compared to controls. This study showed that JZTX-14 inhibited Nav1.5 channels in MDA-MB-231 cells and inhibited cell migration by downregulating *S100A4* and *FBXO2* and upregulating *SERPINB2* (Figure 9).

A peptide with anticancer activity was isolated and purified from animal venom to construct a new anticancer drug library. Compared to small-molecule drugs, peptide drugs have the advantages of high selectivity and affinity for molecular targets, and it is not easy to make tumours resistant to drugs (Wu et al., 2014). To date, many anticancer peptides have been screened from animal venom. For example, the spider polypeptide latroeggtoxin-V inhibits the proliferation, migration, and cell cycle of breast cancer MDA-MB-231 cells in a concentration-dependent manner (Xu et al., 2018). Melittin, the main peptide component of *Apis mellifera* venom, inhibits EGF-induced cell movement and invasion by inhibiting the PI3K/Akt/mTOR signalling pathway in breast cancer cells (Lim et al., 2019). Disintegrins extracted from totonacan rattlesnake (*Crotalus totonacus*) venom showed extracellular matrix (ECM) protein adhesion and migration inhibitory effects in MDA-MB-231 and HMEC-1 cells (Rivas Mercado et al., 2020). *Rhopalurus juncus* scorpion venom inhibits the proliferation of MDA-MB-231 cells and induces cell apoptosis through the mitochondrial apoptosis pathway (Díaz-García et al., 2017). This study provides a new target for inhibiting breast cancer metastasis and identifies a potent natural peptide, JZTX-14, for treating TNBC. With advances in chemical synthesis technology, synthetic active peptides have become increasingly promising candidates for the clinical treatment of tumours.

## Data Availability

The datasets presented in this study can be found in online repositories. The names of the repository/repositories and accession number(s) can be found below: https://www.ncbi.nlm.nih.gov/, SRP407545.
